# Lumbar plexus injury in an anterior fracture dislocation of sacroiliac joint: a case report and review of literature

**DOI:** 10.1007/s11751-013-0177-4

**Published:** 2013-09-17

**Authors:** Narender Kumar Magu, Rohit Singla, Paritosh Gogna, Amanpreet, Nishant Jain, Shalini Aggarwal

**Affiliations:** Pt. B. D. Sharma PGIMS Rohtak, 319/19, Medicos Agencies, Opp. Civil Hospital, Rohtak, Haryana 124001 India

**Keywords:** Lumbar plexus, MRI, Anterior, Dislocation, Sacroiliac, Exploration

## Abstract

Displaced unstable pelvic fractures are commonly associated with disruption of the osteoarticular junction of the sacroiliac joint. Posterior sacroiliac dislocation are commonly reported but there are only few reports the anterior type of sacroiliac dislocation where the iliac bone fractures and displaces anterior to sacrum, often in combination with fractures of pubic rami and symphyseal injuries. We present a case of an anterior type of sacroiliac fracture dislocation which was associated with a lumbar plexus injury involving both motor and sensory components. Preoperative neurological assessment was done by MRI scan. The tented nerve roots were explored and decompressed surgically, and sacroiliac fixation was done after reduction in the fracture and joint.

## Introduction

Unstable pelvic ring injuries are uncommon and are mainly caused by high-energy trauma. They result in extensive disruption of the pelvis and high rates of mortality and late morbidity [1]. Displaced and unstable pelvic fractures are commonly associated with disruption of the osteoarticular junction of the sacroiliac joint [1]. Posterior sacroiliac dislocation occurs in most of the instances but there are few reports only for the *anterior* type of sacroiliac dislocation, wherein the iliac bone fractures and displaces anterior to the sacrum often in combination with fractures of the pubic rami and concomitant symphyseal injuries [2–6].

Neurological injury after pelvic disruption can be a cause of significant morbidity [7, 8]. We present a case report of an anterior type of sacroiliac fracture dislocation, which was associated with lumbar plexus injury involving both motor and sensory components. Stabilization of the posterior sacroiliac ligament complex was done by indirect reduction along with decompression of the stretched lumbar nerve roots.

## Case report

**Fig. 1:**
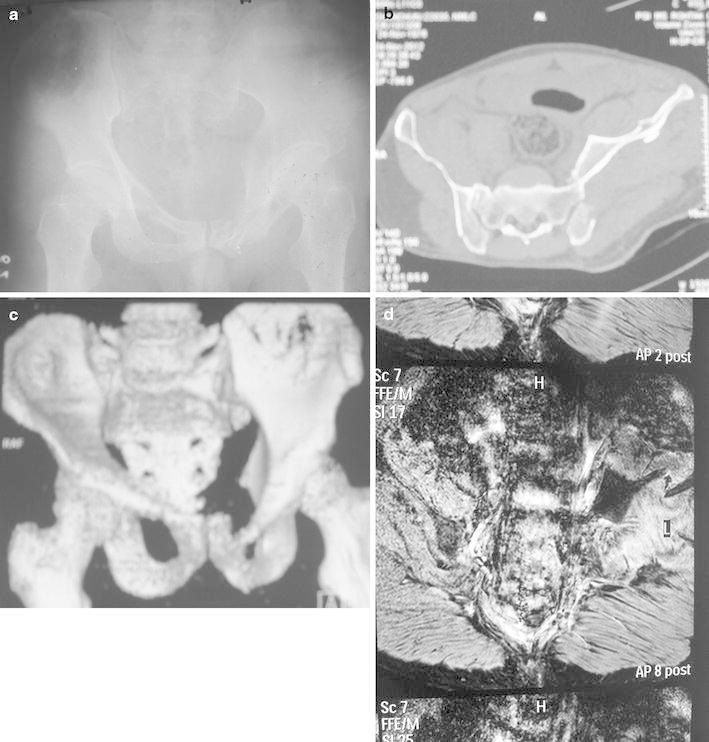
**a** Radiograph showing the sacroiliac fracture dislocation with fractures of bilateral pubic rami. **b** A CT scan (transverse section) showing the anterior dislocation of ilium over sacrum. **c** A CT scan (3D) showing fracture pubic rami with anterior displacement of iliac crest along with part of sacrum. **d** An MRI scan showing integrity of lumbosacral plexus

A 36-year-old male was brought to the accident and emergency department at our university hospital after a road traffic accident. A passenger was sitting on the front seat of a moving three wheeler auto vehicle, the vehicle toppled after a head on collision with a standing heavy utility vehicle. After initial stabilization, a detailed secondary survey was performed. The left lower limb was noted to be externally rotated and short. A Morel Lavallée lesion was present over the left trochanteric and presacral area. Neurological examination showed sensory deficits in the L_4_ and L_5_ dermatomes and motor deficits in the L_1_–L_5_ myotomes (grade 2 power in hip flexors, adductors and knee extensors, and grade 0 power in extensor hallucis longus and ankle dorsiflexors). Plantar flexion of the foot was present; the knee reflex was absent, whereas perianal sensation and the anal reflex present. The initial radiological assessment included antero-posterior, inlet–outlet views, obturator oblique and iliac oblique views of involved hip joint. A sacroiliac fracture dislocation was identified along with fractures of pubic rami bilaterally (Fig. 1a). The lesion was classified an AO/OTA *type C* injury. Computerized tomography (CT) scanning was obtained with 3D reconstruction. This revealed the ilium to be dislocated anterior to the sacrum along with fractures of the iliac plate and pubic rami in a transverse section (Fig. 1b, c). Magnetic resonance imaging, done to evaluate the integrity of the lumbosacral plexus, showed *tented nerve roots* over the fractured ends and sacral ala (Fig. 1d).

**Fig. 2:**
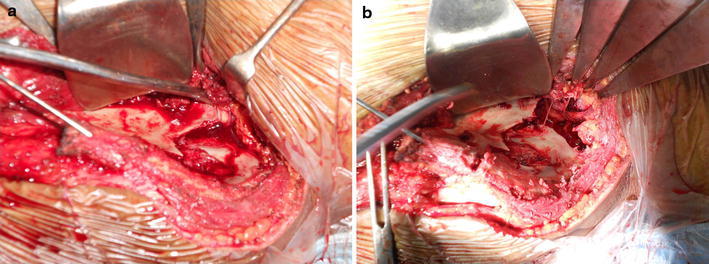
**a** Stretched lumbar nerve roots over the fractured iliac plate. **b** A relaxed nerve root after osteotomy of bone fragment

**Fig. 3:**
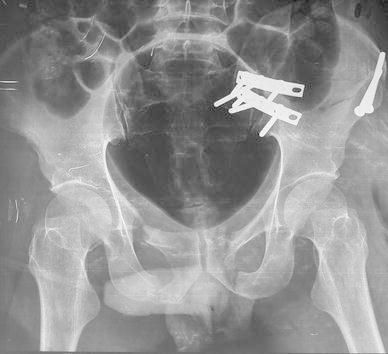
**a** Sacroiliac dislocation fixed with two 3.5-mm narrow DCPs with nerve roots visible

An open reduction and nerve exploration was carried out. The patient was operated under combined spinal–epidural anaesthesia in the supine position. Through an ilio-femoral approach, the iliacus muscle was reflected from the iliac crest; sartorius was divided at the antero-superior iliac spine (ASIS), leaving 1 cm of tendon stump remaining attached to the ASIS. The nerve roots could be identified stretched over the sharp fractured margin of iliac bone close to the lateral articular margin of the sacrum (Fig. 2a). The distal part of the ilium along with its distal articulation with sacrum was displaced anteriorly over the sacrum, putting significant traction on the entire lumbar plexus. It was observed that longitudinal manual traction through the lower limb would cause further damage to the stretched nerve. One centimetre of the fracture fragment was osteotomized to release tension from the stretched nerve (Fig. 2b). The upper part of ilium attached to the sacrum could be identified; the entire plexus could now be traced in front of ala of sacrum, after lifting the tissue subperiosteally. The dislocation was reduced after identifying the sciatic buttress which was found intact. The dislocation was fixed with two 3.5-mm dynamic compression plates (DCPs). The broken ilium was also fixed with two cancellous screws (Fig. 3a). Wound closure was done over double-negative suction drains.

As part of the aftercare, physiotherapy was started and deep venous thrombosis prophylaxis provided. An ankle foot orthosis (AFO) was used. Surgical wound healing was uneventful. Post-operatively, electromyography revealed very few motor unit potentials (MUPs) in left vastus lateralis, vastus medialis, adductor longus, adductor magnus, EHL and gluteus maximus. The medial head of the left gastrocnemius muscle exhibited 30–40 % recruitment, a reduced amplitude and long-duration MUPs. The Hoffmann reflex of soleus on both sides was normal suggesting, possibly, both lumbar nerve roots and the lumbosacral trunk were involved. At last, follow-up at 14 months radiological union was recorded. Clinically, ambulation was pain free and the range of hip flexion was 100°, internal rotation was limited to 20°, and external rotation was full. Neurologically, there was full recovery in hip flexors, adductors and knee extensors. There was no recovery in EHL and ankle dorsiflexion.

## Discussion

The pelvic ring plays many important roles; it accommodates the intra-pelvic organs and bears body weight and provides support to trunk and spinal column. It is, therefore, essential to stabilize and restore the oval-shaped osseo-ligamentous structure of the pelvic ring. Unstable fractures of the pelvic ring are an increasingly frequent outcome of motor vehicle trauma [1, 6]. Sacroiliac disruptions are common among unstable fractures of pelvis. The literature highlights the more commonly posterior type of sacroiliac dislocation and its association with neurological injuries [7–10]. The anterior type of sacroiliac fracture dislocation has been described infrequently by a few authors, most of which have occurred in a paediatric age group [2–6]. The present case is an unusual presentation as the fractured ilium along with the sciatic buttress was found stretching the lumbar plexus and was dislocated anterior to the sacrum. The posterior part of the ilium was found maintaining its continuity with the sacrum.

Neurological injury continues to be a major cause of long-term morbidity after pelvic disruption. Many authors agree that the overall prevalence of neurological injury is related primarily to the presence of posterior ring disruption [8, 11]. Schmal et al. [12] have reported in their multicentric study of 3607 patients that rate of neurological dysfunction increases from 1.5 % in Tile’s type A to 14.4 % in Tile’s type C fractures. Studies have demonstrated that patients with double-vertical pelvic fractures (combined injury to the anterior third of the pelvic ring and the sacroiliac area) are most at risk, with a 46 % incidence of neurological injury [13]. Persistent paraesthesia in the distribution of the lumbar nerve roots has been reported even in long-term follow ups of such pelvic injuries [9].

The diagnosis of neurological injuries is a major problem and can take a backstage in the presence of acute trauma settings [10]. Patients with unstable pelvic ring fractures frequently have associated head, chest or lower extremity injuries and may be unable to cooperate with a thorough clinical examination [7]. Myelography has been used by some authors to better delineate the pattern of neurological injury preoperatively [14, 15]. However, myelographic diverticula observed do not always correspond to the clinical deficit. On initial neurological assessment in the present case, injury to the lumbar plexus nerve roots could be diagnosed. Later EM G studies showed low MUPs in sacral myotomes, indicating lumbosacral trunk involvement. This clinical dilemma has been highlighted by several authors [13, 16]. Lumbar magnetic resonance imaging (MRI) is a promising alternative for preoperative assessment of possible lumbar plexus injury in acute pelvic injury setting. Beaulieu et al. [16] have used MRI scans for neurological assessment of lumbosacral plexus in their case series of 4 patients with pelvic fractures. With this, they were able to decide the approach for surgical exploration, whether trans-peritoneal or trans-sacral. We have used an MRI scan in our case preoperatively and were able to locate tented nerve roots over the sacral ala and fractured margins (Fig. 1d). This would forewarn the surgeon for a more cautious approach while reducing the sacroiliac dislocation and decrease any chances of further iatrogenic nerve injury. Tonetti et al. [10] have reported iatrogenic neurological deficit in 7 of their 44 cases.

Injury to lumbosacral plexus can occur from compression, traction or frank avulsion of nerve roots. Qi Zhang et al. [6] have reported four cases of anterior dislocation of sacroiliac dislocation but have mentioned little about neurological lesions in their cases. Goodell performed surgical exploration of 3 patients with nerve injury complicating fractures of pelvis [15]. In each of their cases, multiple levels of nerve root avulsion were seen. Traction injury may affect nerves as they exit the greater sciatic notch or as they cross the anterior aspect of the sacroiliac joint [17]. We were able to visualize and palpate cord-like stretched nerve roots over fractured fragment while lifting the iliacus muscle sub-periosteally with finger tips. We observed that longitudinal traction to the limb could further stretch the plexus. We therefore performed an osteotomy of the fracture fragment which released the tension (Fig. 2b). We believe it was an appropriate manoeuvre as longitudinal traction could have caused further damage to the plexus.

## Conclusion

Our case highlights the presentation of an anterior type of fracture dislocation of sacroiliac joint in an adult patient and its outcome, wherein we were able to locate the stretched and tented lumbar nerve roots preoperatively with MRI scan and confirm the findings intra-operatively. This enabled an approach and creation of an osteotomy to enable fracture and joint reduction without further damage to the nerves.

## References

[1] Choy WS, Kim KS, Lee SK, Park HJ (2012). Anterior pelvic plating and sacroiliac joint fixation in unstable pelvic ring injuries. Yonsei Med J.

[2] Feinblatt JS, Phieffer LS, Lawyer RB (2010). Anterior sacroiliac dislocation. Orthopedics.

[3] Bouguennec N, Gouin F, Piétu G (2012). Isolated anterior unilateral sacroiliac dislocation without pubic arch disjunction. Orthop Traumatol Surg Res.

[4] Blondel B, Glard Y, Launay F, Jacopin S, Jouve JL, Bollini G (2011). Anterior dislocation of the sacroiliac joint in children: a new technique for pelvic fixation. J Pediatr Orthop B.

[5] Lee DH, Jeong WK, Inna P, Noh W, Lee DK, Lee SH (2011). Bilateral sacroiliac joint dislocation (anterior and posterior) with triradiate cartilage injury: a case report. J Orthop Trauma.

[6] Zhang Q, Chen W, Liu H, Su Y, Pan J, Zhang Y (2009). The anterior dislocation of the sacroiliac joint: a report of four cases and review of the literature and treatment algorism. Arch Orthop Trauma Surg.

[7] Reilly MC, Zinar DM, Matta JM (1996). Neurologic injuries in pelvic ring fractures. Clin Orthop Relat Res.

[8] Langloh ND, Johnson EW, Jackson CB (1972). Traumatic sacroiliac disruptions. J Trauma.

[9] Hersche O, Isler B, Aebi M (1993). Follow-up and prognosis of neurologic sequelae of pelvic ring fractures with involvement of the sacrum and the iliosacral joint. Unfallchirurg.

[10] Tonetti J, Cazal C, Eid A, Badulescu A, Martinez T, Vouaillat H, Merloz P (2004). Neurological damage in pelvic injuries: a continuous prospective series of 50 pelvic injuries treated with an iliosacral lag screw. Rev Chir Orthop Reparatrice Appar Mot.

[11] Majeed SA (1992). Neurologic deficits in major pelvic injuries. Clin Orthop.

[12] Schmal H, Hauschild O, Culemann U, Pohlemann T, Stuby F, Krischak G (2010). Identification of risk factors for neurological deficits in patient with pelvic fractures. Orthopedics.

[13] Conway RR, Hubbell SL (1988). Electromyographic abnormalities in neurologic injury associated with pelvic fracture: case reports and literature. Arch Phys Med Rehabil.

[14] Barnett HG, Connolly ES (1975). Lumbosacral nerve root avulsion: report of a case and review of literature. J Trauma.

[15] Goodell CL (1966). Neurologic deficits associated with pelvic fractures. J Neurosurg.

[16] Beaulieu JY, Oberlin C, Arnaud JP (2008). Surgical management of neurological complication of pelvic girdle rupture. J Bone Joint Surg Br.

[17] Huittinen VM, Slatis P (1971). Nerve injury in double vertical pelvis fractures. Acta Chir Scand.

